# Antimicrobial resistance of bacterial enteropathogens isolated from stools in Madagascar

**DOI:** 10.1186/1471-2334-14-104

**Published:** 2014-02-25

**Authors:** Frederique Randrianirina, Elisoa Hariniana Ratsima, Lova Ramparany, Rindra Randremanana, Hanitra Clara Rakotonirina, Tahiry Andriamanantena, Fanjasoa Rakotomanana, Soatiana Rajatonirina, Vincent Richard, Antoine Talarmin

**Affiliations:** 1 Clinical Biology Center, Institut Pasteur de Madagascar, Antananarivo, Madagascar; 2 Epidemiologic Unit, Institut Pasteur de Madagascar, Antananarivo, Madagascar; 3 Molecular Biology Unit, Institut Pasteur de Madagascar, Antananarivo, Madagascar; 4 Institut Pasteur de Madagascar, Antananarivo, Madagascar

**Keywords:** Antibiotic susceptibility, Bacterial pathogens, Madagascar, Diarrhea

## Abstract

**Background:**

Diarrheal diseases are a major public health problem in developing countries, and are one of the main causes of hospital admissions in Madagascar. The Pasteur Institute of Madagascar undertook a study to determine the prevalence and the pathogenicity of bacterial, viral and protozoal enteropathogens in diarrheal and non-diarrheal stools of children aged less than 5 years in Madagascar. We present here the results of the analysis of antimicrobial susceptibility of the bacteria isolated during this study.

**Methods:**

The study was conducted in the community setting in 14 districts of Madagascar from October 2008 to May 2009. Conventional methods and PCR were used to identify the bacteria; antimicrobial susceptibility was determined using an agar diffusion method for enterobacteriaceae and MICs were measured by an agar dilution method for *Campylobacter sp*. In addition to the strains isolated during this study, *Salmonella sp* and *Shigella sp* isolated at the Pasteur Institute of Madagascar from 2005 to 2009 were included in the analysis to increase the power of the study.

**Results:**

Twenty-nine strains of *Salmonella sp*, 35 strains of *Shigella sp*, 195 strains of diarrheagenic *E. coli*, 203 strains of *C. jejuni* and 71 strains of *C. coli* isolated in the community setting were tested for antibiotic resistance. Fifty-five strains of *Salmonella sp* and 129 strains of *Shigella sp* isolated from patients referred to the Pasteur Institute of Madagascar were also included in the study. Many *E. coli* and *Shigella* isolates (around 80%) but fewer *Salmonella* isolates were resistant to ampicillin and trimethoprim/sulfamethoxazole. A small proportion of strains of each species were resistant to ciprofloxacin and only 3% of *E. coli* strains presented a resistance to third generation cephalosporins due to the production of extended-spectrum beta-lactamases. The resistance of *Campylobacter sp* to ampicillin was the most prevalent, whereas less than 5% of isolates were resistant to each of the other antibiotics.

**Conclusion:**

The highest prevalence of antimicrobial resistance was to ampicillin and trimethoprim/sulfamethoxazole. Antibiotic treatment is not recommended for children with diarrhea in Madagascar and the emphasis should be placed on oral rehydration.

## Background

Diarrheal diseases are a major public health problem in developing countries, especially in Africa [1,2]. More than 1.8 million children under 5 years of age die of diarrheal disease every year [2].

The agents capable of causing infectious diarrhea include a wide variety of bacteria, viruses, and parasites. Treatment includes rehydration and in some cases antimicrobial therapy. The acute diarrheal diseases for which antimicrobial therapy is recommended include shigellosis, campylobacteriosis and infection with *Vibrio cholerae*, *V. parahaemolyticus* and *V. vulnificus*[3,4]. Improving our knowledge of antimicrobial resistance among enteric pathogens is of particular importance in the developing world, where the rate of diarrheal diseases is highest. The continuous increase in antimicrobial resistance among enteric pathogens in developing countries is becoming a serious concern. However, resistance patterns are often region-specific, and are only very poorly documented in Africa. The data available relating to antimicrobial resistance in bacteria responsible for diarrheal disease in Madagascar is limited. Few studies have described resistance patterns of Shigella, and the most recent was more than 20 years ago [5]; we found no reports concerning the antimicrobial resistance *Salmonella*, *Campylobacter* or diarrheagenic *Escherichia coli* in Madagascar.

The Pasteur Institute of Madagascar recently launched a program to determine the prevalence and the pathogenicity of bacterial, viral and protozoal enteropathogens in diarrheal and non-diarrheal stools of children less than 5 years of age, in 14 different districts of Madagascar [6]. Here, we present an analysis of the antimicrobial resistance of pathogens isolated in this study and of enteric pathogens isolated from the stools of patients referred to the Pasteur Institute of Madagascar for diarrheal disease.

## Methods

### Patients enrolled during the multicenter study

**Figure 1:**
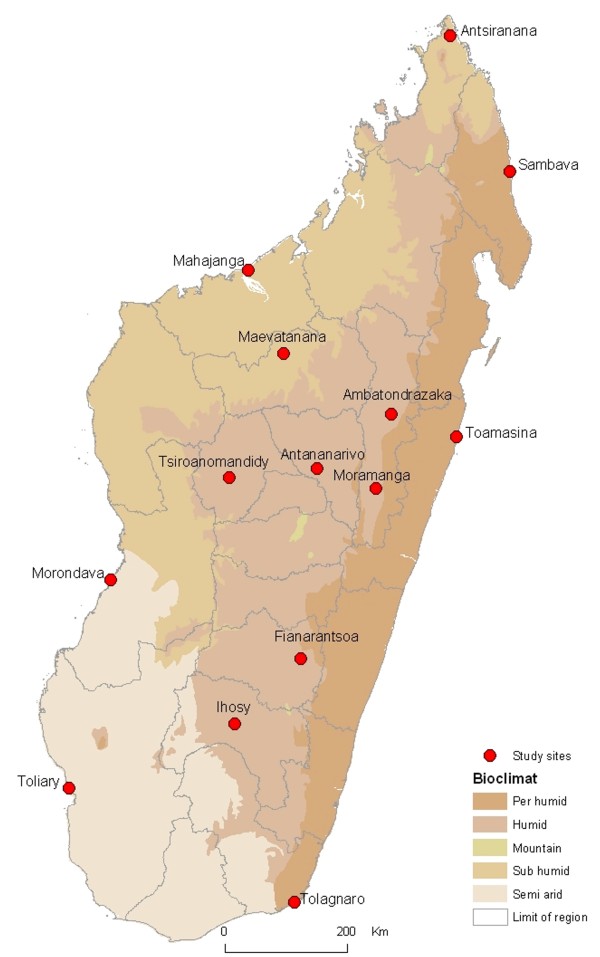
Map of Madagascar with the location of the sites where patients were enrolled during the multicenter study.

The study was conducted in 14 districts of Madagascar as previously described (Figure 1) [6], from October 2008 to May 2009 during the rainy season, for three consecutive weeks at each site. The study was carried out in the community setting, not in hospitals or health centers. A mobile laboratory well equipped (fridge, incubator, Bunsen, microscope), installed in a four-wheel drive truck, was used for the laboratory procedures in optimal field conditions as soon as the stools were emitted. A generator was used to maintain the electricity when the engine was stopped. Temperatures of the fridge and the incubator were controlled using an electronic thermometer. To avoid contamination, stools were processed one by one near the flame of the Bunsen.

Participants were randomly selected from children less than 5 years of age in several villages in each district. Participants provided clinical and epidemiological data and a fecal sample for identification of bacterial, viral and protozoal enteropathogens.

Diarrheic patients were children less than five years of age with acute diarrhea (emission of three or more unformed stools within a 24 h period) without current treatment. After emission of stools, all case patients received treatment according to the World Health Organisation guidelines for diarrheal disease [7].

Non diarrheic subjects were children under five years of age with no history of diarrhea in the previous 3 months and were enrolled at the same time as case subjects (one control for every three cases). Information on demographic and historical clinical data were obtained from parents of both cases and controls and recorded in questionnaires. There was no follow-up after the initial recruitment of patients.

### Patients referred to the Pasteur Institute

Retrospective data concerning the resistance to antibacterial drugs of strains of *Salmonella* and *Shigella* isolated from patients referred to the Pasteur Institute from 2005 to 2009 were analysed in the study to increase the number of strains and give a more complete view of the resistance to antibacterial drugs in these species circulating in Madagascar. For these patients fresh stools were brought directly by the patients or their family to the Pasteur Institute and were processed within 2 hours after reception.

### Laboratory procedures

#### Bacterial isolation and identification

Bacteria, including *Salmonella* spp., *Shigella* species, *Campylobacter* spp and *Escherichia coli*, were isolated using standard microbiological procedures. Stool samples were plated on Hektoen agar for the detection of *Salmonella* and *Shigella*, Eosin Methylene Blue and Uriselect for the detection of *E. coli* and on Karmali agar for the detection of *Campylobacter*. Plates were incubated at 37°C, under specific microaerophilic conditions for *Campylobacter* (campygen, Oxoid, France). During the multicenter study, the plates positive for colonies suspected to be pathogenic *Salmonella spp.*, *Shigella species* or *E. coli* and all Karmali agar plates were sent at least every 2 days to the Pasteur Institute for complete identification. *Campylobacter* spp. were confirmed with the hemagglutination test-kit (Campy dry spot, Oxoid, England), as recommended by the manufacturer.

Colonies typical of *E. coli* on EMB or Uriselect medium were further tested by PCR for genes encoding virulence factors associated with diarrheagenic *E. coli*[8].

#### Susceptibility testing

The antibiotic susceptibilities of all *Salmonella*, *Shigella* and diarrheagenic *E. coli* isolates were determined by a standard antibiotic disk diffusion technique according to the Antibiogram Committee of the French Microbiology Society (CASFM) [9]. The antibiotics tested were nalidixic acid, ciprofloxacin, amoxicillin, amoxicillin + clavulanic acid, ticarcillin, ceftazidime, cefotaxime, cefoxitin, gentamicin and trimethoprim-sulfamethoxazole. Antimicrobial susceptibility was scored using the breakpoint criteria, as defined by the CASFM [9]. Extended Spectrum Betalactamase (ESBL) production was deduced from the resistance phenotype and results of the double-disk synergy test using conventional combinations (cefotaxime, ceftazidime, ceftriaxone *vs*. clavulanic acid) [10].

*Escherichia coli* ATCC 25922, *Staphylococcus aureus* ATCC 25923 and *Pseudomonas aeruginosa* ATCC 27853 were used as controls.

The minimum inhibitory concentrations (MICs) of five antimicrobial agents were determined for *Campylobacter sp*, using agar dilution methods. Two-fold serial dilutions of antibiotics were added to molten Mueller–Hinton II agar (Oxoid) supplemented with 5% sheep blood at a temperature of 37°C. The plates were seeded with 10^4^ cfu/spot of bacteria by means of a multipoint inoculator and incubated at 35°C under CO_2_ for 48 h in a microaerobic atmosphere (Campygen, Oxoid, France).

The antimicrobials evaluated were ciprofloxacin, erythromycin, ampicillin, tetracycline and gentamicin. Reference strains *Campylobacter coli* ATCC 49941 and *Campylobacter jejuni subsp jejuni* ATCC 49943 were used as controls for both susceptibility testing and growth. The MIC breakpoints used were those recommended by the CASFM [9].

### Ethical issues

The national Ethics Committee of Madagascar approved the research protocol. Informed consent was obtained from all the participants either directly or through their legal and competent guardians.

### Statistical analysis

R software (R Foundation for Statistical Computing, Vienna, Austria) was used for data analysis. In univariate analysis, the two-tailed Fisher’s exact test was used to compare categorical variables for antibiotic resistance according to the study site (cross sectional community study or diagnostic activities of the Pasteur Institute of Madagascar). Factors identified by univariate analysis as being associated with p-values <0.20 were retained for multivariate analysis. Differences were considered significant if the p-value was <0.05.

## Results

Demographic characteristics of the patients included in the multicenter study were already given in a previous article; all patients were under 5 years old [6]. Concerning the patients presenting at the Pasteur Institute they were limited to age; the median age was 6 (range: 4 months - 82 years), and patients were divided in two groups (age < 5 years old, n = 81) and patients > = 5 years old, n = 90) for the analyses. Twenty-nine strains of *Salmonella sp*, 35 strains of *Shigella sp*, 195 strains of diarrheagenic *E. coli*, 203 strains of *C. jejuni* and 71 strains of *C. coli,* isolated from diarrheic and healthy children in the community [6], were tested for antibiotic resistance. Fifty-five strains of *Salmonella sp* and 129 strains of *Shigella sp* isolated from patients referred to the Pasteur Institute of Madagascar were included in the study. Among the *Shigella* isolates, 156 could be typed: there were 3 strains of *S. boydii*, 12 strains of *S. dysenteriae*, 99 strains of *S. flexneri* and 42 strains of *S. sonnei*.

Many of the Enterobacteriaceae were resistant to antibiotics widely used in Madagascar; in particular, resistance to penicillin A and trimethoprim-sulfamethoxazole was highly prevalent among *Shigella* and *E. coli* isolates (60% to 80% of strains were resistant; Table 1), but less so among *Salmonella sp* (35.7 of the strains were resistant to amoxicillin and 2.3% to trimethoprim-sulfamathoxazole) (Table 1). There were no significant differences in rates of resistance between the different types of diarrheagenic *E. coli*.

**Table 1:** **Susceptibility of *Salmonella*, *Shigella sp *** **and diarrheagenic *Escherichia coli *** **isolated from stools of patients with acute diarrhea, in Madagascar**

	***Salmonella sp (n = 84)***			***Shigella sp*** **(n = 164)**			**Diarrheagenic** ***E. coli*** **(n = 195)**		
**Antimicrobial**	**Resistant**	**Intermediate**	**Susceptible**	**Resistant**	**Intermediate**	**Susceptible**	**Resistant**	**Intermediate**	**Susceptible**
	**N (%)**	**(%)**	**(%)**	**(%)**	**(%)**	**(%)**	**(%)**	**(%)**	**(%)**
Amoxicillin	30 (35.7)	0 (0.0)	54 (64.3)	103 (62.8)	0 (0.0)	61 (37.2)	160 (82.1)	0 (0.0)	35 (17.9)
Amoxicillin/Clavulanic acid	2 (2.3)	3 (3.6)	79 (94.1)	12 (7.3)	40 (24.4)	112 (68.3)	2 (1.0)	21 (10.8)	172 (88.2)
Ticarcillin	30 (35.7)	0 (0.0)	54 (64.3)	102 (62.2)	1 (0.6)	61 (37.2)	161 (82.6)	0 (0.0)	34 (17.4)
Cephalothin	2 (2.3)	3 (3.6)	79 (94.1)	14 (8.5)	37 (22.5)	113 (69)	6 (3.1)	11 (5.6)	178 (91.3)
Cefotaxime	1 (1.2)	0 (0.0)	83 (98.8)	0 (0.0)	0 (0.0)	164 (100)	6 (3.1)	0 (0.0)	189 (96.9)
Ceftazidime	1 (1.2)	0 (0.0)	83 (98.8)	0 (0.0)	0 (0.0)	164 (100)	3 (1.5)	3 (1.5)	189 (96.9)
Cefoxitin	0 (0.0)	0 (0.0)	84 (100)	0 (0.0)	0 (0.0)	164 (100)	0 (0.0)	1 (0.5)	194 (99.5)
Gentamicin	0 (0.0)	0 (0.0)	84 (100)	0 (0.0)	0 (0.0)	164 (100)	2 (1.0)	0 (0.0)	193 (99.0)
Nalidixic acid	1 (1.2)	2 (2.3)	81 (96.5)	1 (0.7)	0 (0.0)	163 (99.3)	21 (10.8)	0 (0.0)	174 (89.2)
Ciprofloxacin	0 (0.0)	0 (0.0)	84 (100)	0 (0.0)	0 (0.0)	164 (100)	6 (3.1)	0 (0.0)	189 (96.9)
Trimethoprim/Sulfamethoxazole	2 (2.3)	0 (0.0)	82 (97.7)	131 (79.9)	5 (3.0)	28 (17.1)	165 (84.6)	2 (1.0)	28 (14.4)

Resistance to ampicillin and ticarcillin was significantly more prevalent among *Salmonella* strains isolated from patients referred to the Pasteur Institute of Madagascar (29 of 55 strains, 52.7%) than among *Salmonella* strains isolated in the community (one of 29 strains, 3.5%) (p < 0.01) (Table 2). Resistance to third generation cephalosporin was found only in one of 84 *Salmonella* strains (1.2%) and six of 195 of diarrheagenic *E. coli* (3.1%); all these resistant strains presented a ESBL phenotype (synergy between third generation cephalosporins and clavulanic acid and susceptibility to cefoxitin).

**Table 2:** **Percentage of isolates expressing resistance for *Salmonella sp*, *Shigella flexneri *** **and *S. sonnei*, according to the type of patient inclusion**

**Species**	**Isolated from (patient age)**	**Number of strains**	**Amx**		**AMC**		**CF**		**TIC**		**SXT**	
			**N**	**%**	**N**	**%**	**N**	**%**	**N**	**%**	**N**	**%**
*S. flexneri*	PIM* (> = 5 years)	34	29	(85)	18	(53)	17	(50)	29	(85)	24	(71)
	PIM (<5 years)	39	36	(92)	25	(69)	25	(69)	36	(92)	30	(77)
	Transversal Study (<5 yrs)	26	23	(88)	6	(23)	6	(23)	23	(88)	26	(100)
	p-value		0.63		0.04		0.05		0.63		<0.01	
*S. sonnei*	PIM (> = 5 years)	26	2	(8)	0	(0)	0	(0)	2	(8)	21	(81)
	PIM (<5 years)	13	1	(8)	0	(0)	0	(0)	1	(8)	13	(100)
	Transversal study (<5 yrs)	3	2	(67)	0	(0)	0	(0)	2	(67)	3	(100)
	p-value		0.03		——		——		0.03		0.17	
*Salmonella sp*	PIM (> = 5 years)	29	11	(38)	1	(3)	1	(3)	11	(38)	0	(0)
	PIM (<5 years)	26	18	(69)	4	(15)	4	(15)	18	(69)	2	(8)
	Transversal study (<5 yrs)	29	1	(3)	0	(0)	0	(0)	1	(3)	1	(3)
	p-value		<0.01		0.04		0.04		<0.01		0.30	

*PIM: Pasteur Institute of Madagascar.

Significant differences in rates of resistance were also found between *S. flexneri* isolated from patients referred to the Pasteur Institute of Madagascar (n = 73) and *S. flexneri* isolated in the community (n = 26) concerning trimethoprime-sulfamathoxazole (74% vs. 100% of strains being resistant, respectively, p < 0.01), nalidixic acid (57.5% vs. 23%, respectively, p < 0.01) and amoxicillin/clavulanic acid (58.9% vs. 23% respectively, p < 0.01) (Table 2). There were differences in rates of resistance between *S. flexneri* and *S. sonnei* for amoxicillin and ticarcillin (88.9% vs. 11.9% of strains were resistant, respectively, p < 0.01), amoxicillin/clavulanic acid (49.5% vs. 0%, respectively, p < 0.01) and cephalotin (48.5% vs. 0%, respectively, p < 0.01). The only difference that persisted in multivariate analysis was the type of patient included in the study: at the Pasteur Institute of Madagascar or in the community (Tables 2 and 3). Among the patients referred to the Pasteur Institute of Madagascar, there was no difference between patients aged less than 5 years and those aged more than 5 years, except for resistance to ampicillin and ticarcillin in *Salmonella*; resistance to these drugs was more prevalent among *Salmonella* isolates from patients younger than 5 years old (P = 0.04) (Table 2).

**Table 3:** **Percentage of susceptible and intermediately susceptible (in brackets) isolates of the *Shigella *** **genera**

**Pathogens (No tested)**	**AMX**	**AMC**	**TIC**	**CF**	**FOX**	**CTX**	**CAZ**	**NA**	**CIP**	**GM**	**SXT**
*Shigella species* (8)	37.5	75 (25)	37.5	75 (25)	100	100	100	87.5	100	100	0 (12.5)
*S. dysenteriae* (12)	66.7	91.7 (8.3)	66.7	91.7 (8.3)	100	100	100	100	100	100	33.3
*S. flexneri* (99)	11.1	50.5 (37.4)	11.1 (1)	50.5 (37.4)	100	100	100	100	100	100	19.2 (4)
*S. boydii* (3)	66.7	100	66.7	100	100	100	100	100	100	100	0
*S. sonnei* (42)	88.1	100	88.1	100	100	100	100	100	100	100	11.9

There was a significant difference between the prevalence of resistance to ampicillin of *C. jejuni* and *C. coli* (66.5% vs. 90.1%, respectively, p < 0.01, OR = 4.6, 95%CI [1.9-12.5]) (Table 4). The rates of resistance to other antibiotics, including tetracycline but excluding nalidixic acid, were low (Table 4).

**Table 4:** **Susceptibility of *Campylobacter sp *** **isolated from stools of patients with acute diarrhea, in Madagascar**

	***C. jejuni***						***C. coli***					
	**Susceptible**		**Intermediate**		**Resistant**		**Susceptible**		**Intermediate**		**Resistant**	
	**n**	**%**	**n**	**%**	**n**	**%**	**n**	**%**	**n**	**%**	**n**	**%**
Erythromycin	160	(78.8)	41	(20.2)	2	(1.0)	50	(70.4)	18	(25.4)	3	(4.2)
Ampicillin	68	(33.5)	95	(46.8)	40	(19.7)	7	(9.9)	36	(50.7)	28	(39.4)
Gentamycin	202	(99.5)	1	(0.5)	0	(0.0)	71	(100.0)	0	(0.0)	0	(0.0)
Tetracycline	201	(99.0)	0	(0.0)	2	(1.0)	70	(98.6)	0	(0.0)	1	(1.4)
Ciprofloxacin	199	(98.0)	2	(1.0)	2	(1.0)	67	(94.4)	0	(0.0)	4	(5.6)

In the multicenter study in the community, there was no significant difference in the rates of resistance to any antibiotic between isolates from cases and those from controls for any species, nor between isolates from different sites.

## Discussion

Antibiotics are often used only as a supplemental tool to treat diarrhea, with oral rehydration being the main treatment. However, in developing countries where many children are malnourished, it may be advisable to use all tools to minimize the duration of the illness and avoid severe dehydration. In most areas of developing countries, bacterial identification is not feasible and hence the antibiogram for each patient cannot be determined. Therefore, extensive knowledge of the prevalence of antibiotic resistance among the various species involved would be extremely valuable. Our study presents the strength of having been conducted in 14 different sites in Madagascar (Figure 1). It is therefore representative of the whole country, at least for diarrheagenic *E. coli* and *Campylobacter sp*. We chose also to include *Salmonella* and *Shigella* strains isolated at the Pasteur Institute of Madagascar to increase the scope of the study. This may have introduced a bias, but any such bias is probably minor because many of patients presenting at the Pasteur Institute of Madagascar for diarrhea had traveled in the country immediately before the onset of symptoms such that the strains isolated did not all originate from Antananarivo. The age of patients is also different but differences are mainly between patients presenting at the Pasteur Institute, whatever their age, and those enrolled in the transversal study.

The prevalence of resistance of *Shigella* and *E. coli* to ampicillin and cotrimoxazole were very high, and much higher than those of *Salmonella sp*. They were similar to the rates of resistance of *E. coli* isolated from urinary tract infections in a previous study [11]. These rates of resistance to ampicillin and cotrimoxazole are also similar to those observed in Botswana and Kenya, where the differences in rates of resistance between *Salmonella* and *Shigella* have also been reported [12,13]. During a previous survey conducted in 1988–1989 in Madagascar, rates of resistance of *Shigella* were different from those we report here [5]. Resistance of *S. dysenteriae* to ampicillin and cotrimoxazole were slightly more prevalent in the previous study (69% vs. 63% and 38% vs. 0%, respectively) whereas resistance of *S. flexneri* to penicillin A and cotrimoxazole have become considerably more prevalent since 1989 (13% vs. 89% and 4.3% vs. 80%, respectively). In our study, no resistance to ciprofloxacin was found in *Salmonella* or *Shigella* isolates, in contrast to what has been described elsewhere, for example on the Andaman Islands [14]. However, according to the CASFM, strains of *Salmonella* resistant or intermediately resistant to nalidixic acid should be categorized as resistant to fluoroquinolones [9]. Therefore three strains in our study should be considered as resistant to ciprofloxacin.

Nevertheless, few of the diarrheagenic *E. coli* isolates were resistant to ciprofloxacin (3.1%).

ESBL-producing *E. coli* were detected but the rates were lower than those of rectal carriage of ESBL-producing *E. coli* observed both in community settings in Madagascar (6.4%) [15], and in children on admission to a pediatric unit in Antananarivo (10%) [16]. This rate was, however, similar to what was observed for *E. coli* isolated from urinary tract infections in the community [11]. The rates of ESBL-producing *E. coli* were also lower than that observed in Cairo, Egypt (14.3%) [17], but higher than in South America where rates of resistance to ampicillin and cotrimoxazole are high whereas no resistance to third generation cephalosporin has been observed in Brazil or Peru [18,19].

Rates of resistance among *Campylobacter sp* were similar to that found in 2005 for isolates from chickens in Antananarivo [20]. The only major difference was that resistance to erythromycin was less prevalent in our (5/274, 2%) than the previous study (18.3% of resistant strains).

The resistance of *Campylobacter sp* to antibiotics frequently used for therapy in veterinary medicine in many countries (tetracycline and ciprofloxacin) was much lower in our study than reported in most other studies [21-24]. It is likely that antibiotics are too expensive for most farmers in Madagascar, and this would explain the susceptibility of most strains in the country.

Rates of resistance were similar in diarrheic and healthy children which is not surprising since strains were probably clonally related in a village. This is also compatible with the fact that the prevalence rates were not different for all these strains between diarrheic and healthy children [6].

In a previous article, we described the types of pathogens detected in this study, and reported that intestinal pathogens were the most frequently encountered. *Giardia lamblia, Trichomonas intestinalis and Entamoeba histolytica* were the only pathogens that were detected at a higher frequency in patients with diarrhea than in patients without diarrhea [6]. These results and those of our antimicrobial susceptibility study argue strongly for a reasoned use of antibiotics to treat diarrhea in children in Madagascar. Indeed, other than *Salmonella*, which are rarely isolated and for which antibiotics are not recommended, the most common enterobacteria (*Shigella* and *E. coli*) isolated from such patients are resistant to penicillin A and cotrimoxazole in nearly four of every five cases. The only available antibiotic that can be administered orally and likely to be effective is ciprofloxacin which is not recommended in children.

## Conclusions

Our study shows a high prevalence of antimicrobial resistance to ampicillin and trimethoprim/sulfamethoxazole. These results and the fact that parasites are frequently responsible for diarrhea in Malagasy children imply that antibiotic treatment is not recommended for children with diarrhea in Madagascar. Therefore, we believe that emphasis should be placed on the importance of oral rehydration in the management of diarrhea in children in Madagascar.

## Competing interests

The authors declare that they have no competing interests.

## Authors’ contribution

FR, ERH, LR, RR, SR participated in the field study and the patients recruitment. FR, ERH, LR, HCT, TA, FR and AT paricipated in the antibiotic susceptibility testing. VR realised the statistical analysis. FR, ERH, VR ansd AT participated in the study design. FR, ERH, LR, VR and AT participated to the redaction of the article. All authors read and approved the final manuscript.

## Pre-publication history

The pre-publication history for this paper can be accessed here:

http://www.biomedcentral.com/1471-2334/14/104/prepub
